# The information that patients, their families, and medical staff wish to know about postoperative pain and its management. Results of the survey after mixed surgical procedures

**DOI:** 10.3389/fpain.2026.1728929

**Published:** 2026-03-02

**Authors:** Dusica Stamenkovic, Maša Petrović, Dragana Unic Stojanovic, Milos Novovic, Dragana Radovanovic, Nebojsa Ladjevic, Aleksandra Jukic, Emilija Dubljanin Raspopovic, Nemanja Rancic, Milijana Miljkovic, Sulin Bulatovic, Boris Bulatovic, Winfried Meissner, Ruth Zaslansky

**Affiliations:** 1 Medical Faculty of Military Medical Academy, University of Defense, Belgrade, Serbia; 2 Department of Anesthesiology and Intensive Care, Military Medical Academy, Belgrade, Serbia; 3 Center of Excellence, Institute for Cardiovascular Diseases “Dedinje”, Belgrade, Serbia; 4 University of Belgrade Faculty of Medicine, Belgrade, Serbia; 5 Department of Anesthesiology and Resuscitation, General Hospital Prijepolje, Prijepolje, Serbia; 6 Oncology Institute of Vojvodina, Sremska Kamenica, Serbia; 7 Urology Clinic, Medical School University of Belgrade, Belgrade, Serbia; 8 Department of Anesthesiology, National Cancer Research Center of Serbia, Belgrade, Serbia; 9 Clinic for Orthopedics Surgery and Traumatology, University Clinical Centre of Serbia, Belgrade, Serbia; 10 Centre for Clinical Pharmacology, Military Medical Academy, Belgrade, Serbia; 11 Department of Anesthesiology and Intensive Care, University Hospital Jena, Jena, Germany

**Keywords:** acute pain, pain education, pain management, patient communication, patient perspective

## Abstract

**Background:**

Informing patients and families about perioperative pain management is a fundamental element of care. However, patients and their families are often not involved developing such resources. Thus, their viewpoints on content, timing, and methods of presentation remain unknown. Our survey aimed to assess perioperative pain information content, timing, and presentation methods most valued by patients, their families, and medical staff.

**Methods:**

The survey took place as part of a multi-center, quality improvement study in 6 hospitals. On the first postoperative day, patients, family members or friends, and medical staff were asked to complete a questionnaire addressing content about pain, side effects of analgesics, the timing of providing the information, and the preferred method of receiving the information. A composite score was created for questions regarding information content.

**Results:**

A significant difference in total composite score of information items about pain characteristics and management (*p* < 0.05) was observed between the groups. No significant difference was observed between groups for questions evaluating side effects of analgesics (*p* = 0.099). A significant difference was observed between patients and family members for questions concerning information post-discharge (*p* = 0.037).

**Conclusion:**

We found that healthcare providers and patients differ in the some aspects of postoperative pain management they consider important. Patients and families were interested in post-discharge pain management, while medical staff rated pain characteristics and management in immediate period the highest. For all groups, the preferred time for providing information about perioperative pain was before surgery at the pre-operative clinic by an anesthesiologist or surgeon.

## 1 Introduction

Providing patients and families with information about postoperative pain management is a fundamental element of care (1). The American Pain Society recommends that the clinicians provide patients and their family, individually tailored, education that includes information on options for managing postoperative pain and document the plan and goals of care. This is a strong recommendation, yet, the quality of the evidence is low (2, 3). Schug and colleagues state in the Acute Pain Management: Scientific Evidence that patients and their caretakers will have greater control over the quality of their pain relief if they are informed regarding treatment efficacy and any adverse effects (4). The positive effects of preoperative educational interventions on pain have been found to reduce opioid use (5, 6) and improve psychological outcomes, particularly with regard to anxiety and personalization of medical care (7, 8).

During our literature review, we found that patient resources (9, 10) have generally been compiled by healthcare providers, with little or no input from patients (3). This was remarkable as it is, in fact, contrary to current standards whereby patient and public involvement is a core principle for developing high-quality evidence-based clinical practice guidelines (11). The principle standard of patient involvement is based on the premise that resources which involve patients and the public will improve the relevance of the outcomes and quality of the decisions that are made (11, 12). Patient and public involvement aims to shift providers from doing something to or for patients, where the health care provider's perspective is dominant, to doing something with them in partnership (11, 13–16).

In a study of patients undergoing outpatient surgery, patients gave the highest priority to information addressing how to care for the pain and side effects once they were home (17). On the other hand, addressing addiction was given the lowest priority (17). In a follow up study interviewing patients whose primary language was not English, findings were similar and surgical subtype, health status, and age did not affect the results (18). Patients after spine surgery expressed interest in establishing realistic expectations about the surgery as a part of the pre-operative process and preparing a comprehensive pain management plan, which included family and social support, after surgery (19). These studies did not explore optimal timing or preferred delivery modality.

The aims of our survey were to: (1) investigate the information that patients, their families and medical staff wish to know about pain and its treatment options in terms of (i) content (ii) presentation and (iii) timing perioperatively; (2) evaluate whether there is a commonality in the information sought after by patients and by family members; (3) evaluate differences about information preferences between patients and medical staff responsible for their care.

We hypothesized that in terms of content, patients in our sample will be similar to those interviewed by Kastanias et al., in that they would like to receive information on how to address their pain and side effects once discharged (17). The information preference on pain management at home is expected to be particularly valued by family members who might be acting as patient advocates. We hypothesized that medical staff will opt for information about pain management delivered on the ward, consistent with routine clinical practice.

## 2 Methods

### 2.1 Survey design and setting

This survey was part of a larger multi-center, multi-national quality improvement (QI) study (Ethical Committee MMA No. 8/2020 date 04.08.2020) (20). Research teams from 6 hospitals in one country (Republic of Serbia) participated in this survey during 3 months.

### 2.2 Participants

The sample included patients who underwent mixed surgical procedures, requiring overnight hospital stay. Inclusion criteria were: (i) providing consent for participation; (ii) age ≥18 years old; (iii) on the first day after surgery (POD1) and on the ward for at least 6 h, and (iv) ability to read in the native language. For family and/or friends present at the time patients were approached, consent was given orally. The medical staff sample consisted of consultant anesthesiologists and surgeons, anesthesiology and surgery residents, and nurses on the ward, who gave oral consent. Since this was an internal anonymous audit performed on a voluntarily basis, no consent was needed from medical staff and family members in line with local regulations at the time of survey performance.

### 2.3 Components of the questionnaire

The original tool “The Information Needs Questionnaire—Pain and Pain Management” (INQPP) was developed based on clinical guidelines, and analyzed and edited by the multidisciplinary committee [Pain Management Education Team (PMET)] dedicated to pain management education (17). PMET consisted of 22 physicians and nurses' experts in the pain field and “allied health professionals” from pharmacy and physiotherapy (17). The tool was tested by postsurgical patients during their hospitalization “for comprehensiveness of content areas and usability” [Kastanias (17), p. 24]. The initial version of the tool was corrected to increase sensitivity by changing the response format from a 5-point to 10-point Likert scale (17).

The original INQPP consisted of 19 questions grouped into 3 sections: (A) pain characteristics and management; (B) side effects of pain medications, and (C) management of pain and side effects once discharged from the hospital (17). We adapted the INQPP, with permission, for use in our patient population and modified the question ratings to an 11-point Likert scale from 0-not important to 10-extremly important. The adapted questionnaire underwent forward translation and back-translation from English to Serbian. Questions were added to assess preferences regarding timing (section D) and methods of presentation (section E) using dichotomous yes/no answers. The adapted questionnaire was then piloted on the national population in 9 patients-family member dyads in postoperative period (Supplementary file). No formal validation of the original or the adapted version of questionnaire was performed.

### 2.4 Data collection

An investigator, who was not involved in the patient's clinical care, approached participants on the first postoperative day (POD1) to provide background regarding the survey and oral guidance on how to complete the questionnaire. The patients were provided with a printed version of questionnaire and left to complete the questionnaire independently without the investigators involvement. In each center, we aimed for a convenience sample, ≥20 patients, matched to a family member or friend. Medical staff consisted of anesthesiologists, surgeons, residents and medical nurses directly engaged in medical care of patients treated on the surgical wards and were invited to complete the survey. However, the medical staff on the ward did not participate as investigators in the survey to overcome potential researcher bias. No target number for staff members and percentage of kinds of professionals that participated was made.

### 2.5 Data analysis

Results are presented as count (%) or mean ± standard deviation, and 25th to 75th percentile range, depending on the data type and distribution. The evaluations reported by the groups were compared using one-way ANOVA, chi-squared, and independent samples t-test, depending on the type of data and analysis. All *p*-values less than 0.05 were considered significant. Data were analyzed using SPSS 29.0 (IBM Corp. Released 2022. IBM SPSS Statistics for Windows, Version 29.0. Armonk, NY: IBM Corp., USA).

#### 2.5.1 Ranking

Items 1 through 19 in the questionnaire were summed to obtain a composite score which was subsequently compared between the groups. Separate composite sub-scores were calculated for the 3 sections addressing pain (items 1–9), side effects of pain medications (items 10–16), and post-discharge (items 17–19). The scores were averaged by question for all groups (i.e., patients, family member, staff), and later ranked from lowest to highest, where higher scores indicate a greater level of importance to the individual completing the questionnaire. We color coded the rankings using conditional formatting in Excel, where a green background indicates the most important, while a red background indicates the least important information, with the midpoint, marked in yellow, being the 50th percentile.

## 3 Results

### 3.1 Participants

**Figure 1:**
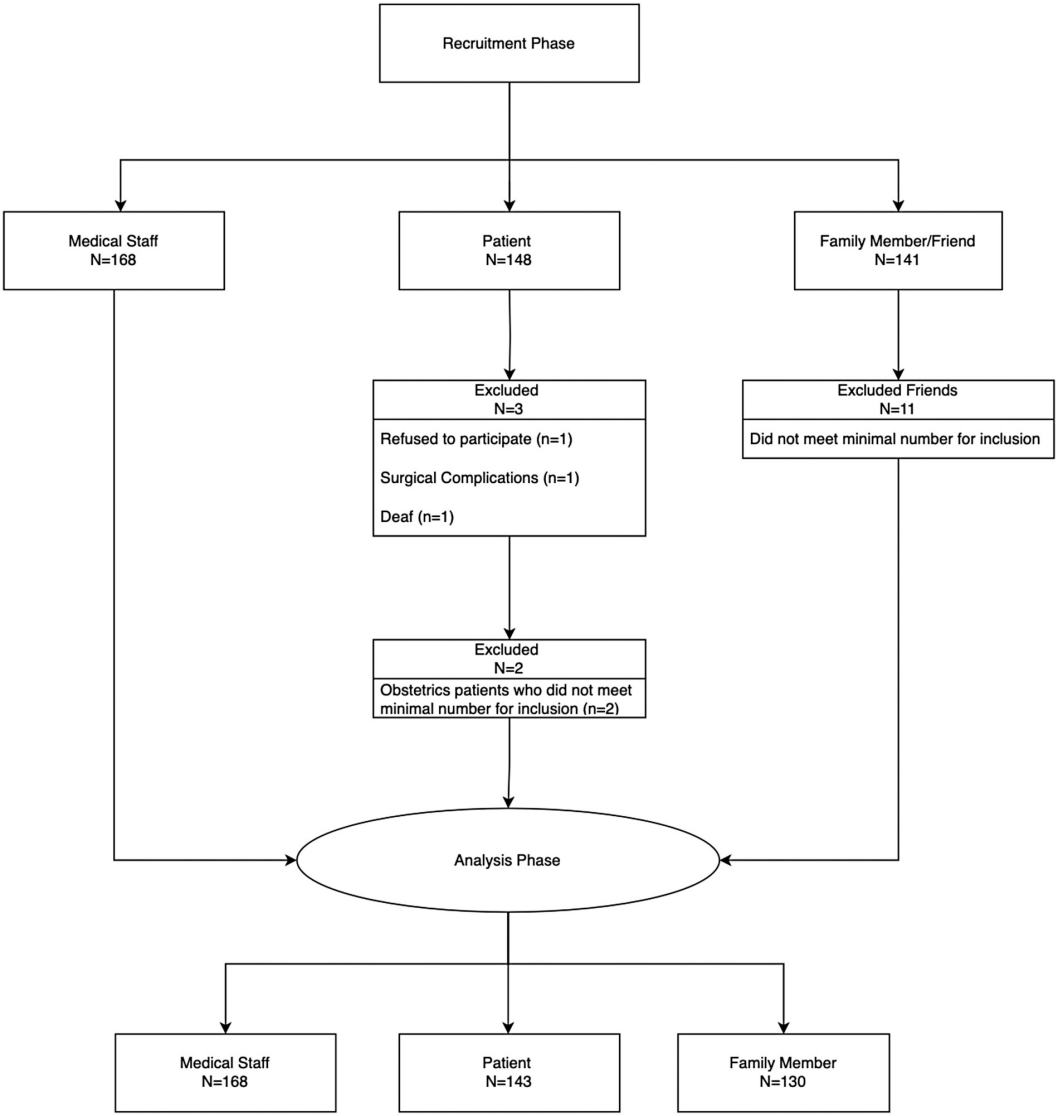
Participant flowchart.

Of the subjects enrolled between August 2019 and March 2020, data from 143 patients, 130 family members or friends of the patient and 168 members of staff from 12 wards and 6 hospitals, were analyzed (Figure 1). The demographic characteristics of the study sample are presented in Table 1. Participants completed the survey in 10–15 min.

**Table 1:** General characteristics of the study population.

Demographics	Patient	Family Member	Medical Staff
Sex (Male)	69 (48.3%)	51 (39.2%)	47 (28.0%)
Age	59.2 ± 15.6	49.9 ± 13.1	38.2 ± 10.4
Highest Level of Education			
Elementary	17 (13.5%)	9 (6.9%)	0 (0%)
High School	81 (64.3%)	66 (50.8%)	99 (58.9%)
College/University	45 (35.7%)	55 (42.3%)	69 (41.1%)
Marital Status			
Single	10 (7.0%)	12 (9.2%)	64 (38.1%)
Married	110 (76.9%)	110 (84.6%)	91 (54.2%)
Divorced	8 (5.6%)	5 (3.8%)	12 (7.1%)
Widowed	15 (10.5%)	3 (2.3%)	1 (0.6%)
Employment Status			
Full time	50 (35.0%)	81 (62.3%)	166 (98.8%)
Part time	4 (2.8%)	3 (2.3%)	2 (1.2%)
Self Employed	5 (3.5%)	7 (5.4%)	0 (0.0%)
Retired	61 (42.7%)	21 (16.2%)	0 (0.0%)
Unemployed	22 (15.4%)	11 (8.5%)	0 (0.0%)
Student	1 (0.7%)	7 (5.4%)	0 (0.0%)
Type of Surgery			
General	50 (35.0%)	N/A	N/A
Orthopedic	20 (14.0%)	N/A	N/A
Obstetrics	10 (7.0%)	N/A	N/A
Urology	20 (14.0%)	N/A	N/A
Cardiovascular	43 (30.1%)	N/A	N/A
Self-reported health status			
Poor	7 (4.9%)	5 (4.5%)	2 (1.2%)
Fair	49 (34.5%)	21 (18.9%)	37 (22.0%)
Good	71 (50.0%)	51 (45.9%)	63 (37.5%)
Excellent	15 (10.6%)	34 (30.6%)	66 (39.3%)
Persistent Pain (yes)	28 (24.8%)	5 (5.2%)	15 (9.9%)

All values are presented as mean ± SD or *n* (%).

### 3.2 Content of information

#### 3.2.1 Pain (pain duration, treatment, whom to speak to)

The one-way ANOVA revealed that there was a statistically significant difference for content of information for the total composite score (ANOVA, *F* = 5.661, *P* = 0.001) and for the composite score of items 1–9 (ANOVA, *F* = 10.835, *P* < 0.001). Following these significant one-way ANOVA a *post-hoc* Tukey Honest significant difference (HSD) test was performed to identify which group means differed significantly. The test revealed that patients had significantly lower total sum scores compared to family members [mean difference = −16.223, 95%CI (−28.46, −3.99), *p* = 0.004], and staff [mean difference = −16.208, 95%CI (−27.7, −4.72), *p* = 0.002]. These results suggest patients rated postoperative pain information lower than family members and staff.

Information about pain intensity (item 1) and duration of postoperative pain (item 2) were ranked significantly lower among patients compared to medical staff and family members (rank 10 vs. 1 vs. 7, respectively) (Table 2). Items 6 and 7 regarding addiction to pain medication, common concerns about pain (item 8) and pain medicine and financial resources for analgesics (item 9) were among the lowest ranked between the three groups (Table 2). Similar findings were observed in the rankings by family and medical staff. For family members, the least important question was regarding financial resources (item 19), and for medical staff the least important was regarding common concerns about pain (item 8).

**Table 2:** Descriptive statistics for each question (Q1–19) in sections A, B and C (information content) of the questionnaire. Results are presented as means and 95% CI [Rank] for mean and *p*-values when comparing means between the three groups. We ranked the questions from 1 (most important) through 19 (least important) based on the mean scores for each question. The coloring was done using conditional formatting in Excel, where a green background indicates the most important, while a red background indicates the least important information, with the midpoint, marked in yellow, being the 50th percentile. Composite scores are presented as mean ± standard deviation.

Questions	Patient	Family	Medical Staff
A) Info about pain			
1. How much pain to expect?	7.3 (6.7–7.9) [10]	8.4 (8.0–8.9) [7]	9.2 (9.0–9.5) [1]
2. How long I can expect to experience pain after surgery?	7.7 (7.2–8.3) [5]	8.6 (8.2–9.0) [5]	9.1 (8.8–9.4) [2]
3. Whom to speak to if I experience pain?	8.0 (7.5–8.5) [4]	8.7 (8.3–9.1) [4]	8.6 (8.3–9.0) [5]
4. The best words to use to explain my pain	5.9 (5.3–6.5) [14]	7.0 (6.5–7.5) [13]	7.5 (7.07–8) [13]
5. How my pain would be treated?	7.6 (7.1–8.2) [6]	8.3 (7.8–8.7) [8]	8.5 (8.1–8.9) [7]
6. If I can get addicted to medications used to treat pain?	5.5 (4.8–6.1) [17]	6.7 (6.1–7.3) [16]	7.1 (6.6–7.6) [15]
7. Other ways of dealing with my pain in addition to medicines	5.4 (4.8–6.1) [18]	6.9 (6.3–7.5) [14]	7.2 (6.7–7.7) [14]
8. Common concerns many patients have about pain and pain medicines	5.2 (4.6–5.9) [19]	6.7 (6.1–7.3) [17]	6.3 (5.8–6.8) [19]
9. If I can get help to pay for pain medicines once I am discharged	5.5 (4.8–6.2) [16]	6.3 (5.7–7.03) [19]	6.8 (6.2–7.3) [17]
Composite Score (Items 1–9)^a^	58.14 ± 23.48	67.62 ± 18.06	70.27 ± 16.85
B)Information about the side effects of pain medications			
10. Which side effects I am most likely to experience?	7.5 (6.9–8.1) [9]	8.51 (8.01–9.01) [6]	7.79 (7.3–8.2) [10]
11. Which are possible side effects, even the rare ones?	5.7 (5.1–6.4) [15]	6.8 (6.2–7.3) [15]	6.7 (6.2–7.1) [18]
12. How likely I was to get the side effects?	6.2 (5.6–6.8) [13]	6.6 (6.1–7.2) [18]	6.9 (6.4–7.4) [16]
13. Who should I speak to if I had side effects?	7.6 (7.1–8.2) [7]	8.1 (7.6–8.6) [9]	8.2 (7.9–8.6) [8]
14. What side effects I should report to staff caring for me?	7.2 (6.6–7.7) [11]	8.1 (7.6–8.6) [10]	7.9 (7.5–8.4) [9]
15. How long might side effects last?	7.1 (6.6–7.7) [12]	7.9 (7.4–8.4) [12]	7.7 (7.2–8.1) [12]
16. How might side effects be treated?	7.5 (6.9–8.1) [8]	8.0 (7.5–8.5) [11]	7.7 (7.3–8.2) [11]
Composite Score (Items 10–16)	48.80 ± 20.99^b^	54.04 ± 17.65^b^	52.89 ± 17.60
C)Information after leaving the hospital			
17. The plan for which medications to take and when	8.7 (8.3–9.2) [1]	9.2 (8.8–9.5) [3]	8.8 (8.4–9.2) [3]
18. Who to call if my pain is not well controlled?	8.7 (8.3–9.1) [2]	9.3 (9.0–9.5) [1]	8.6 (8.3–8.9) [6]
19. What can do if I still have pain or side effects?	8.7 (8.3–9.1) [3]	9.2 (8.9–9.5) [2]	8.7 (8.4–9.1) [4]
Composite Score (Items 17–19)	26.06 ± 7.01^b^	27.57 ± 4.74^b^	26.04 ± 5.94
Total Composite Score (Items 1–19)^a^	133.00 ± 45.93	149.22 ± 35.33	149.21 ± 36.17

A *p*-value < 0.05 was considered statistically significant.

^a^ Statistically significant difference identified by one-way ANOVA.

^b^ Statistically significant difference identified by an independent-samples t-test.

#### 3.2.2 Side effects of pain medications (duration, treatment, whom to speak to)

With regard to questions 10–16, evaluating side effects of pain medication, we did not observe a statistically significant difference between groups (*F* = 2.103, *P* = 0.099). However, when comparing only patients and family members, there was a statistically significant difference observed (*F* = 2.103, *P* = 0.026). Furthermore, we found that patients and medical staff were more interested about who to refer to in the presence of side effects (item 13, rank 7 and 8, respectively), while family members, on the other hand, were more interested about the type of side effects (rank 6).

#### 3.2.3 Information important after leaving the hospital

Items in this section (questions 17–19) were of greatest importance for patients especially knowledge about the plan for analgesia type and timing (item 17) (Table 2). When further comparing patients to family members, we observed a statistically significant difference with regard to the questions concerning information after leaving the hospital, questions 17–19 (*t* = 2.096, *P* = 0.037). The patients prioritized the plan about pain management (item 17), while family member ranked information about person responsible for information if pain is “not well controlled” (item 18) as number 1 (Table 2).

### 3.3 Timing of providing the information

With regard to timing of providing information about perioperative pain (item 20), overall, the preferred time for providing information about perioperative pain was before surgery at the pre-operative clinic (Table 3). The second choice was different among the groups. Patients and medical staff opted for getting information after surgery, on the first day, while family members tended to prefer “before surgery, as a leaflet sent to my home” (Table 3). There was a significantly higher number of patients compared to family members and medical staff who expressed that they do not wish to have information about pain (Chi-squared, *p* = 0.001).

**Table 3:** Section D (item 20) on timing of providing information about perioperative pain with using dichotomous yes/no answers. We present the number of patients who answered yes (*n*) and the percentage. The percentage exceeds 100, as more than one answer was allowed. All values are presented as *n* (%).

Question 20 and answers	Patient	Family Member	Medical Staff
20.When would you have liked to receive the information? (yes)			
Before surgery at the pre-operative clinic	99 (62.3%)	112 (87.5%)	163 (96.4%)
Before surgery, as a leaflet sent to my home	54 (37.8%)	61 (47.3%)	66 (39.3%)
After surgery, on the first day	58 (40.6%)	59 (45.7%)	92 (54.8%)
Any time after surgery	55 (38.5%)	55 (44.0%)	73 (43.4%)
I do not wish to have this sort of information	31 (21.7%)	23 (17.8%)	14 (8.3%)

### 3.4 Preferred method of receiving information about pain

The preferred methods for receiving information (item 21) is shown in Table 4. All three groups, gave the highest preference to receiving the information orally by the anesthesiologists, followed by the surgeon and then nurse. The second most preferred option for patients and family members was a leaflet left on the ward, while for medical staff the second most preferred option was oral information presented by a nurse.

**Table 4:** Section E (item 21) address the preferred method of receiving information using dichotomous yes/no answers. We present the number of patients who answered yes (*n*) and the percentage. All values are presented as *n* (%). Multiple responses were allowed for this question.

Question 21 and answers	Patient	Family Member	Medical Staff
21. How would you like to receive the information? (yes)			
Orally by an Anesthesiologist	99 (69.2%)	103 (79.2%)	161 (95.8%)
Orally by a Surgeon	93 (65.0%)	97 (74.6%)	120 (71.4%)
Orally by a Nurse	79 (55.2%)	71 (54.6%)	109 (64.9%)
As a booklet which I can find on the ward	82 (57.3%)	73 (56.2%)	62 (36.9%)
As a poster in the ward	67 (46.9%)	55 (42.3%)	45 (26.8%)
As a leaflet sent to my phone	50 (35.0%)	46 (35.4%)	41 (24.4%)
As a video sent to my phone	38 (26.6%)	39 (30.0%)	31 (18.5%)
As a video in closed circuit TV on the ward	58 (40.5%)	56 (43.4%)	40 (23.8%)

## 4 Discussion

This survey identified differences between patients, their families, and medical staff regarding information needs on postoperative pain and its treatment. Patients and families prioritized information about pain management after discharge, while medical staff emphasized information about pain intensity and duration. Most participants preferred receiving information during the preoperative clinic visit. However, preferences for the second-best timing varied: patients and staff preferred the first postoperative day, while families favored “before surgery, as a leaflet sent to home.” Across all groups, the preferred format was verbal information delivered by an anesthesiologist or surgeon.

### 4.1 Content of information

The content of the information provided is of the utmost importance. Irrelevant content information in concurrence with the vulnerable state of patients, particularly in conjunction with nonfamiliarity of the hospital environment may lead to increased patient stress and anxiety (21, 22). Although we as medical staff have constant communication with patients that does not necessarily mean that we are giving them the information of their interest (3). All groups were interested in knowing the person to whom they should speak with if they experience pain, which suggests that patients rely on communication with medical staff as this offers patients the possibility of taking an active role in their pain management (3, 23).

Patients rated information about postoperative pain duration and treatment during hospitalization lower than family members and staff, highlighting a potential area for further investigation in patient support mechanisms (3). Most patients and their family members ranked the questions regarding information after leaving the hospital as the most important. Similar findings were reported in previous studies (17, 18). Medical staff should be encouraged to provide patients and their families with realistic expectations for the recovery process which includes pain management (19); however, the lack of education for this type of conversation is noticeable in practice (3, 16).

Of note is that addiction to analgesics was of low interest for all participants in our survey similar to Kastanias (17), Kastanias (18). However, patients expressed concerns regarding medication side effects after discharge from hospital which was similar to studies that explored opioid analgesic use during that period (24). The difference regarding perception of opioid addiction during the immediate postoperative period and post-discharge period might be the result of treatment duration i.e., short opioid application for postoperative pain management while patient is in the hospital. The literature has also discussed the possibility that information on pain treatment could raise patients' expectations for pain reduction, potentially leading to a higher demand for postoperative opioids (25, 26). The question remains whether our results indicated a greater confidence in receiving appropriate medical care and the belief that pain will be treated with adequate medications when necessary, thus, sparing opioid use (20) or if our results simply reflect lack of awareness toward the addiction in the population we studied. A recently published review concluded that limited data are available about adult patients “perceptions and experiences” with opioid use during surgery and the immediate postoperative period (27). Therefore, additional investigations are recommended as patient perceptions and experience have an influence on opioid prescription (27). Apart from opioids, the awareness about side effects related to non-opioid analgesics and adjuvants if used for longer periods of time, must be addressed to patients.

### 4.2 Timing of providing the information

Providing information at the pre-operative clinic gives patients the opportunity to think and prepare, and it is therefore unsurprising that all groups preferred for this timing. In our survey, patients and medical staff preferred receiving information on the first day after surgery as a second option, while family members tended to receive information before surgery, as a leaflet sent to their home. Education and providing information to patients should be considered as a continuous process, and not just be limited to the preoperative period.

Surprisingly, a sizable portion of patients (21.7%) and family members (17.8%) in our survey opted that they do not wish to receive information. This might be a consequence of patients not identifying their role and its function in pain management, as was noticed in qualitative interpretive phenomenological research in patients from single cardiac rehabilitation center by Walton at al (28). However, the patients presented this role through different terms including the discussion of the subjective nature of pain and the importance of using a pain assessment tool (28), which may similarly reflect the desire for perioperative pain-related information, found in our study. Additionally, ignoring deeper knowledge about pain might be culturally related to the belief that surgery is supposed to be painful and that they will deal with the pain. This phenomenon has been previously described as the “positive social appraisal of ability to cope with pain” [Eshete (29), p.10), which may stem from patients being unaware of their situation and thus disregarding the information they receive, either because they do not fully understand it or due to fear of being perceived as “weak” by society for acknowledging their pain. The concept of patients' medical ignorance or their “right not to know” (Davies, 2020, p.300) was recently discussed, suggesting that in stressful situations, such as awaiting surgery or a diagnosis, information might be distressingly overwhelming for patients (30). However, this data necessitates further exploration so that medical professionals can be properly guided.

### 4.3 Preferred method of receiving information about pain

While our surveyed groups preferred orally delivered information, other cohorts opted for a combination of oral and written forms (23, 31). The difference in our patients' opinion and results from other studies might be due to cultural differences. Both forms of information presentation have their benefits and shortcomings. Providing information orally facilitates interactive communication between patients and the medical staff (“face to face”) and can be more effective in improving postoperative pain outcomes compared to video presentations (7). However, receiving information orally might be overwhelming and difficult to memorize in potentially stressful circumstances such as when waiting for surgery, while the written form offers a format that can be referred to repeatedly (23). Additionally, if the information is delivered in the preoperative clinic, patients have time to absorb and process the information, offering the opportunity to ask additional questions. Similarly, to other studies, our participants found physicians i.e., anesthesiologist as a valuable source of information (3, 23).

### 4.4 Survey limitations

Our study has several limitations. The first limitation is that study took place in single country, and therefore cultural differences in how pain is perceived and managed, as well as healthcare practices, could limit the generalizability of the findings to other settings or countries. However, our study offers valuable information regarding patients' and family members' needs in middle income countries, particularly that patients from middle- and low-income countries are less frequently presented in pain outcome standardization initiatives (11). Secondly, the survey relied on reports by patients and their families without including information from patient's medical records and we were, therefore, unable to evaluate associations with factors such as reasons for surgery (e.g., cancer-related vs. not or acute vs. chronic condition) which might influence the nature of information that patients request.

The third limitation is the lack of medical staff breakdown in types or years of experience in our survey might impact the survey results, therefore we advocate for future studies to explore this topic. The fourth limitation worth noting is the exclusion of pharmacists from the survey. While the role of pharmacists varies between countries, many of the questions relate to side effects and pain management after discharge—areas where pharmacists could play an important role.

The participants were asked about their information needs and preferences after the surgery on POD1, and patients' experiences may change over time. It is worth noting that participating patients were fully alert and willing to take part in the survey, they also filled the questionnaire without assistance. Additionally, we relied on a convenience sample which may not fully represent the broader population of postoperative patients, leading to potential biases in the findings. For example, patients who volunteered to participate might have different experiences of pain (i.e., less pain) or attitudes than those who did not. To the best of our knowledge there is no formally validated questionnaire for exploration of patients' needs regarding information on postoperative pain management. For our survey we adapted the original INQPP tool, which had only been used in surgical contexts in previous studies (17, 18). With development of core outcome sets for acute pain which includes pain (intensity and duration), pain influence on physical functioning, quality of recovery and analgesics use (11) there is a need to develop a validated tool for assessing patients' needs regarding information on postoperative pain management, that will enable the comparison over healthcare settings and populations.

### 4.5 Future directions

We suggest that future studies should include additional parameters in the evaluation such as presence of comorbidities, preoperative diagnosis, and history of previous surgeries. Furthermore, it is important to take into consideration the country's economic level and type of healthcare system model (i.e., national health insurance vs. private) as there could be a difference in findings.

The findings of this study could contribute to development of educational programs for healthcare providers on effective communication with patients and their families regarding pain and its management. Future directions should focus on improving methods for information transfer by actively involving patients as partners in the co-creation of educational materials that are understandable to both patients and their family members (32). At this point during the patient information development phase, priority should be given to continuous perioperative information about pain with information given predominantly in the preoperative clinic preferably by an anesthesiologist or surgeon. None of the previous studies evaluated the time needed to provide patients and family information about postoperative pain. This is noteworthy as information presentation, which is of the highest importance to patients, proper timing, and methods might streamline delivery. Future studies should assess the generic topics of interests in patients to aid in the further development of a more applicable education materials.

## 5 Conclusion

Our findings support that in relation to information needs following a surgical experience, patients are most interested in pain management after hospital discharge. While the majority of respondents expressed a strong desire for perioperative pain-related information, a subset of patients reported a limited interest in receiving such information, highlighting the need for further investigation into factors influencing informational preferences. Across all study groups, the preferred timing for the initial presentation of information was the preoperative clinic, most commonly delivered by an anesthesiologist or surgeon. Although patients and their family members generally favored in-person communication, our findings support that written materials should also be made available.

Our survey demonstrates that the focal point of medical staff differs from patients', therefore, we still need to learn more about this topic. Availability of such knowledge will enable development of a conversation model dedicated to postoperative pain information and interaction between the patient, their family, and medical staff as a tool for improving clinical practice.

## Data Availability

The original contributions presented in the study are included in the article/Supplementary Material, further inquiries can be directed to the corresponding author.
