# Supplementary material for: The information that patients, their families, and medical staff wish to know about postoperative pain and its management. Results of the survey after mixed surgical procedures
Source: Front Pain Res (Lausanne). 2026 Mar 2;7:1728929. doi: 10.3389/fpain.2026.1728929 (PMC12990131; doi:10.3389/fpain.2026.1728929)
Supplement: Supplementary file 1 [file Supplementaryfile1.docx]

Supplementary Material

# Supplementary Figures and Tables

**Contents**

| **S-1** | Modification of the questionnaire used in the survey | **3** |
| --- | --- | --- |
| **S-2** | Modified questionnaire English version | **5** |
| **S-3** | Modified questionnaire in the study population’s national language version | **7** |

**S-1. Modification of the questionnaire used in the survey**

Participants received a questionnaire which was based on The Information Needs Questionnaire - Pain and Pain Management (INQPP) tool developed by Kastanias et al. for use with day surgery patients in Canada (Kastanias). The questionnaire was translated and adapted from English to study population’s national language and piloted for use in patients from Serbia. Adaptations was performed based on our clinical experience and contacts with patients during pilot study by team members (DS, RZ, MN) and presented in the Supplementary material. Copyrights of original questionnaire were obtained from the publisher through Copyright Clearance Center (01907764). The changes in INQPP included:

**1)Additional three questions with multiple choice answers in section C of the questionnaire:**

Q20.When would you like to receive the information (you may select several answers)?

Q21. How would you like to receive the information (you may select several answers)?

**2) Additional questions with multiple choice answers in section E of the questionnaire:**

Are you a patient /family member / friend of patient;

Q30.Did you have a persistent painful condition for 3 months or more before coming into hospital for this surgery?

Q31.Did you find the number of questions in this questionnaire acceptable or were there too many?

**3)We excluded the following questionnes since we considered them inrelevant for our population:**

Q23.What country were you born?

Q24. What language do you mainly speak?

Q28.What is /was your occupation?

Q29.What city /town do you live in or is closest to you?

Q33.Where did you have pain?

Q35.How do you learn best?

Q36. How often do you use the Internet at home?

Q37.How often do you use the Internet to find health information?

Q38. Have you seen the United Health Network web site?

Q39. Would use the United Health Network web site for health information?

The final version in English and study population’s national Language of Modified The Information Needs Questionnaire - Pain and Pain Management (INQPP) tool developed by Kastanias et al. for use with day surgery patients in Canada (Kastanias).

Kastanias P, Denny K, Robinson S, Sabo K, Snaith K What do adult surgical patients really want to know about pain and pain management? Pain Management Nursing 2009; 10: 22-31. (The original Questionnaire courtesy of Pain Management Nursing through Copyright Clearance Centre license number 01907764).

**S-2. Modified questionnaire English version**

**What do patients and their families wish to know about pain and its treatment in relation to their surgery?**

We would like your opinion as to which information you, as a patient, and a family member of yours, would like to receive about pain and its treatment in relation to your surgery.

We also wish to know the format in which you would like to receive this information.

Circle the number from 0-10 that best describes how important it would have been for you to receive this information. ‘0’ means that the information item is not important to you at all, ‘5’ means it is moderately important to you and ‘10’ means that the information item is extremely important to you.

**A. Information about pain**

**I would have liked to know:**

1. how much pain to expect 0 1 2 3 4 5 6 7 8 9 10

2. how long I can expect to experience pain after surgery 0 1 2 3 4 5 6 7 8 9 10

3. who to speak to if I experience pain 0 1 2 3 4 5 6 7 8 9 10

4. the best words to use to explain my pain 0 1 2 3 4 5 6 7 8 9 10

5. how my pain would be treated 0 1 2 3 4 5 6 7 8 9 10

6. if I can get addicted to medications used to treat pain 0 1 2 3 4 5 6 7 8 9 10

7. other ways of dealing with my pain in addition to medicines 0 1 2 3 4 5 6 7 8 9 10

8. common concerns many patients have about pain and pain medicines 0 1 2 3 4 5 6 7 8 9 10

9. if I can get help to pay for pain medicines once I am discharged 0 1 2 3 4 5 6 7 8 9 10

**B. Information about the side effects of pain medicines**

**I would have liked to know:**

10. which are the side effects I am most likely to experience 0 1 2 3 4 5 6 7 8 9 10

11. which are possible side effects, even the rare ones 0 1 2 3 4 5 6 7 8 9 10

12. how likely I was to get the side effects 0 1 2 3 4 5 6 7 8 9 10

13. who I should speak to if I had side effects 0 1 2 3 4 5 6 7 8 9 10

14. which side effects I should report to staff caring for me 0 1 2 3 4 5 6 7 8 9 10

15. how long side effects might last 0 1 2 3 4 5 6 7 8 9 10

16. how side effects might be treated 0 1 2 3 4 5 6 7 8 9 10

**C. Information after leaving the hospital**

**I would like to know:**

17. The plan for which medications to take and when 0 1 2 3 4 5 6 7 8 9 10

18. Who to call if my pain is not well controlled 0 1 2 3 4 5 6 7 8 9 10

19. What I can do if I still have pain or side effects 0 1 2 3 4 5 6 7 8 9 10

**D. Timing of receiving information about pain**

**20.When would you have liked to receive the information (you may select several answers)?**

1.Before surgery at the pre-operative clinic YES / NO

2.Before surgery, as a leaflet sent to my home YES / NO

3.After surgery, on the first day YES / NO

4.Any time after surgery YES / NO

5.I do not wish to have this sort of information YES / NO

**E. Method of receiving information about pain**

**21.How would you like to receive the information (you may select several answers)?**

| 1. Orally by an Anesthetist | YES / NO |
| --- | --- |
| 2. Orally by a Surgeon | YES / NO |
| 3. Orally by a Nurse | YES / NO |
| 4. As a booklet which I can find on the ward | YES / NO |
| 5. As a poster in the ward | YES / NO |
| 6. As a leaflet sent to my phone | YES / NO |
| 7. As a video sent to my phone | YES / NO |
| 8. As a video in closed circuit TV on the ward | YES / NO |
| 9. Other (please write) |  |

**22. What other information you would have liked to receive in relation to pain and its management?**

_________________________________________________________________________________

_________________________________________________________________________________

**F. About you**

Are you a patient /family member / friend of patient (please circle)

23. **You are a?** Female_____ Male____

24. **What year were you born?** _____________

25. Are you single / married / divorced / widower

26. **What is the highest education you have?**

Elementary school_____________ High school _____________ College/university___________

27. **What is your employment status?** Full time___ Part time___ Self-employed___ Retired___ Not employed___ Student___

28. **What type of surgery did you / member of your family come to the hospital for? General surgery \ orthopedic \ obstetrics \ urology**

29. **In general, how would you rate your own health?** Poor____ Fair____ Good____ Excellent ___

30.Did you have a **persistent painful condition for 3 months** or more before coming into hospital for this surgery? YES / NO

31.Did you find the number of questions in this questionnaire acceptable or were there too many? Acceptable / Too many (select your choice)

If there are too many questions, please list the ones you would wish to remove.

**S-3. Modified questionnaire in the study population’s national language version**

**Информације о болу након операције које интересују пацијента и чланове његове породице**

**Шта Ви као пацијент желите да знате о болу након операције?**

**Шта Ви као породица желите да знате о болу након операције члана Ваше породице?**

Поштовани,

Желимо да чујемо Ваше мишљење, које информације о болу и његовој терапији пре и након операције желите да добијете.

Такође нас интересује формат у коме желите да добијете ове информације.

Молимо Вас да заокружите број од 0 до 10 који најбоље описује значај информације коју желите да добијете везано за бол, а која би Вам била саопштена пре операције „**0**“ означава да информација није значајна за Вас, а „**10**“ значи да је информација од изузетног значаја за Вас.

**А) Информације о болу**

**Желим да знам следеће:**

1. Колику јачину бола могу да очекујем?0 1 2 3 4 5 6 7 8 9 10

2. Колико дуго ће трајати бол након операције?0 1 2 3 4 5 6 7 8 9 10

3. Коме да се обратим уколико осећам бол?0 1 2 3 4 5 6 7 8 9 10

4. Речи којима ћу моћи да опишем свој бол?0 1 2 3 4 5 6 7 8 9 10

5. Како ће се лечити мој бол?0 1 2 3 4 5 6 7 8 9 10

6. Да ли могу постати зависан/а од лекова који се примењују у терапији бола након операције?0 1 2 3 4 5 6 7 8 9 10

7. Који су други начини терапије бола поред лекова?0 1 2 3 4 5 6 7 8 9 10

8. Шта најчешће брине пацијенте када је у питању бол и лекови који се користе у терапији болa?0 1 2 3 4 5 6 7 8 9 10

9. Да ли здравствено осигурање покрива трошкове лекова који се користе у терапији бола након операције, када одем кући? 0 1 2 3 4 5 6 7 8 9 10

**Б) Информације о нежељеним ефектима лекова који се користе у терапији бола**

**Желим да знам следеће:**

10. Који су нежељени ефекти лекова у терапији бола који се

најчешће могу јавити?0 1 2 3 4 5 6 7 8 9 10

11. Који су нежељени ефекти који се могу јавити, укључујући и

оне који су ретки?0 1 2 3 4 5 6 7 8 9 10

12. Колика је вероватноћа да ћу имати неки нежељени

ефекат лека против болова?0 1 2 3 4 5 6 7 8 9 10

13. Коме да се обратим уколико се појави

нежељени ефекат лекова?0 1 2 3 4 5 6 7 8 9 10

14. Који нежељени ефакат треба да пријавим

медицинском особљу?0 1 2 3 4 5 6 7 8 9 10

15.Колико дуго може трајати нежељени ефекат?0 1 2 3 4 5 6 7 8 9 10

16.Како се могу лечити нежељени ефекти?0 1 2 3 4 5 6 7 8 9 10

**В) Информације о болу које желим да сазнам везано за период након напуштања болнице**

**Желим да знам следеће:**

17. План узимања лекова против бола, назив лека и

време примене0 1 2 3 4 5 6 7 8 9 10

18. Кога да позовем уколико мој бол није добро

контролисан0 1 2 3 4 5 6 7 8 9 10

19. Шта да учним уколико имам болове

и поред терапије или нежељене ефекте 0 1 2 3 4 5 6 7 8 9 10

20. Када би желели да добијете информацију? (можете изабрати више опција)

1. Пре хируршког захвата у анестезиолошкој амбулантиДА / НЕ

2. Пре хируршког захвата, брошура послата на моју кућну адресуДА / НЕ

3. Након хируршког захвата, првог дана од операцијеДА / НЕ

4. У било које време након хируршког захватаДА / НЕ

5. Не желим да добијем информације о болуДА / НЕ

21. Како желите да Вам се саопште информације о болу? (можете изабрати неколико одговора**)**

1. Усмено од стране анестезиологаДА / НЕ

2. Усмено од стране хирургаДА / НЕ

3. Усмено од стране медицинске сестреДА / НЕ

4. Као брошура коју ће ми дати на одељењуДА / НЕ

5. Као постер на одељењуДА / НЕ

6. Као брошура послата на мој мобилни телефонаДА / НЕ

7. Као видео клип послат на мој мобилни телефонДА / НЕ

8. Као видео презентација коју могу одгледати на ТВ уређају у

болесничкој соби ДА / НЕ

22. Које су остале информације које бисте желели да имате о болу након операције, а које нису покривене питањима у овој анкети?

**_________________________________________________________________________**

**_________________________________________________________________________**

**Г) О Вама**

Да ли сте: пацијент / члан породице / пријатељ (заокружите одговор)

23. Пол? Женски_____ Mушки_____

24. Које године сте рођени? _____________

25. Да ли сте: сами / у браку / разведени / брачни партнер је преминуо

26. Које је Ваше образовање? Основна школа_______________

Струковна школа/ гимназија_______________ Виша школа/Факултет_______________

27. Подаци о запослењуПуно радно време______ Пола радног времена______

Самостално запослени ______ У пензији______ Незапослени ______ Студирате______

28. Који оперативни захват је рађен у Вашем случају? Абдоминална хирургија / Ортопедија / Породиљство **/** Урологија (заокружите одговор)

29. Како би сте оценили Ваш здравствени статус? Слаб ______ Коректан______

Добар______ Одличан______

30. Да ли сте имали бол које је трајао три или више месеци

након хируршког захвата? ДА / НЕ (заокружите одговор)

31.Да ли мислите да је број питања у овој анкети прихватљив или имa

превише питања?Прихватљив / Превише питања (заокружите одговор)

Уколико има превише питања молимо да напишете која су то питања
